# Creating Care for People Who Self-Harm through Transformation of Aesthetic Objects

**DOI:** 10.1007/s10912-025-09941-w

**Published:** 2025-04-21

**Authors:** Veronica Heney

**Affiliations:** Institute for Medical Humanities, Durham University, Durham, UK

**Keywords:** Care, Narratives, Self-harm, Interpretation, Reading practices

## Abstract

The role of fiction in enabling care for people who self-harm is primarily framed as a relation of protection through absence or avoidance. It is frequently suggested that fiction should avoid depicting self-harm, lest it encourage readers to begin self-harming, framing those who self-harm as passive and in need of protection. This paper will demonstrate that when the perspectives of people who self-harm are centred in the analysis of texts and their effects, the practice of reading and viewing fiction emerges as a more active, creative, and relational experience, which brings self-harm close rather than holding it at a distance. Indeed, such active engagement through material practices like zine-making, event attendance, and repeated viewing of a singular scene is understood as that which makes care possible. Through a novel interdisciplinary approach, this brings together sociological and literary methods to explore the dynamic relation between a text and its effects. Drawing on both qualitative interviews with people with experience of self-harm and close readings of creative texts including the Showtime TV series *The L Word* (2004–2009) and Andrea Gibson’s poem ‘I Sing The Body Electric, Especially When My Power’s Out’ (2011), the paper traces the complex relation between texts and the care they make possible. Thus, I extend existing theorisations of care as intimate and relational to the context of self-harm. Specifically, I outline the way in which care is not predetermined, singular, and universal, and explore the ways that a relation of care can be invited by aesthetic qualities.

## Introduction

The role of fiction in enabling care for people who self-harm is primarily framed as a relation of protection through absence or avoidance — that fiction should avoid depicting self-harm, lest it encourage readers to begin self-harming. Psychologists Janis Whitlock, Amanda Purlington, and Marina Gorschkoich draw on script theory to argue that when self-harm is depicted as “painless, effective, and common, inhibition may be lowered and scripts which support its value adopted” (2009, 152). Literary scholar Kimberley Reynolds critiques young adult novels which rely on dramatic irony to “teach young readers what not to do” because this “risks normalising self-harm” and leading readers to self-harm themselves (2007, 9). Care is understood as silence and as distance from self-harm through avoidance, and texts are primarily framed as translucent containers for a self-evidently harmful conceptual object, which reading or viewing transfers wholesale into the lives of anyone consuming the text. This is a paternalistic framing, wherein self-harming readers and viewers are passive vessels, and all active engagement with a text (and with the idea of self-harm) is dangerous or harmful (Heney, 2024; McWade, 2019).

This paper will demonstrate that centring perspectives of people who self-harm reveals reading and viewing fiction as an active, creative, and relational experience, which brings self-harm close through deep attention, emotional engagement, and transformative material practices. This encourages an assessment of the fictional or creative texts connected to self-harm that moves beyond an examination of their content (Gray 2017; Miskec & McGee 2007). It posits as an area of emergent study the active, transformative use which people who self-harm make of creative texts as sites of care, and the aesthetic properties through which texts invite this engagement.

To explore the interconnection between texts and the ways they are experienced by people who self-harm, I will take an interdisciplinary approach, using sociological and literary studies methods. The data used in this paper is based on my doctoral research in which I interviewed 17 people with experience of self-harm regarding its representation in fiction; the study received ethical approval from the University of Exeter Department of Psychology. Self-harm was defined as “an act, normally a repeated, habitual act, which in some way causes direct harm to the body but one where the focus and purpose of the act is this harm itself and not some other goal” (Steggals 2015, 9). This definition resonated with participants, particularly in its separation of self-harm from attempted suicide, although this distinction was understood as blurry. Participants referenced a range of forms of self-injury, and also connected their experiences to other broadly self-destructive behaviours, such as excessive alcohol consumption or disordered eating. This paper takes up their sense of self-harm as a category that was both capacious and meaningfully specific.

## Research methods

Fourteen participants were white, white British, white Scottish, white Irish, white Jewish, or white European; one participant was mixed race, one was Eurasian, and one was British Asian. Nine participants were in their mid-teens to early 20s, two participants were in their late 20s, five were in their 30s, and one was in her 60s. Eleven participants described themselves as either queer (5), bisexual (3), pansexual (1), lesbian (1) or asexual (1), while 4 participants described themselves as straight or heterosexual. All participants were paid for their time and received a copy of their transcript that they could edit, and were contacted to feedback on the analysis of the data, following accepted good practice (Faulkner 2004).

In both my doctoral thesis and this paper, interview data were analysed using Natasha Mauthner and Andrea Doucet’s (1997) extension of Lyn Brown and Carol Gilligan’s Listening Method (1991), which involves a process of multiple, differently focused readings and encourages the researcher to respond to participants “both emotionally and intellectually” (Woodcock 2010, 148). I adapted the focused sequential readings; I first identified key areas of content (such as overall assessments of fictional texts), then noted the analytic frameworks participants drew on (such as authenticity or glamourisation), and next focused on connections (whether similarities or contrasts) between transcripts. Finally, I noted my own responses following Mauthner and Doucet’s recommendation that the researcher “reads for herself in the text” (197, 126).

Creative texts as objects of care was not explicitly included within my research questions, and so was not a topic I asked directly about. Thus, this was not a dominant theme throughout the 17 interviews and in drawing together these examples I do not argue that they represent a complete or definitive picture of the topic. Rather they function as a starting point. In attending to points of connection between different participants, these sections of data stood out to me in the way they reflected a material, active engagement with creative texts, rather than an assessment of their content. This paper seeks not to answer or resolve the question of care and creativity in relation to self-harm, but to open up new avenues of consideration and future exploration. Specifically, I suggest that in addition to studying the significance of medical treatment, in both fiction and life (as I have done elsewhere, Heney 2024; Make Space 2023), we might beneficially attend to the affective dimension of care. Thus, this paper offers a collection of case studies organised thematically, providing compelling glimpses into the complex topic of self-harm’s relation to textual, creative objects of care. It raises (rather than answers) questions about what care means or should be in the context of self-harm.

Through this analysis, care emerges as dynamic and relational — not as a form of paternalistic protection, whose meaning and desired outcomes are easily established. The paper specifically explores dimensions of care such as affect, engagement, and attention. In understanding such practices and experiences as care, I am informed by the work of feminist scholars — I note that philosopher Joan Tronto defines the values of caring as “attentiveness, responsibility, nurturance, compassion, [and] meeting others’ needs” (1993, 2). In this work, I concentrate on the significance of attentiveness and compassion — in doing so I am not arguing for their primacy, nor articulating a universal theory of care, but rather elucidating a key facet of what care might be in relation to self-harm. Throughout, my exploration of care is underpinned by the work of Donna Haraway, whose theorisation of care as ‘staying with’ thorny or difficult subjects speaks to the significance of closeness which I identify in the data (2016). I draw out ‘closeness’ as an intertwinement of affective and material practices, whereby actions such as zine-making or repeated viewing are accompanied by a broader emotional shift, making it possible to engage with the topic, concept, and experience of self-harm without the shame, disgust, and othering that participants so frequently encountered and experienced. These experiences of closeness are an under-studied area in relation to self-harm, where work on care primarily focuses on material practices of medical treatment, institutionalised inpatient care, and cessation. Thus the paper contributes to theorisations of care which critique dominant, neoliberal framings of care as functioning at a distance and anonymously (as laid out by anthropologist Lisa Stevenson, 2014), to instead explore care’s intimacy and sociality, beyond medicalised frameworks and settings.

In this paper, I interweave exploration of the interview data with analysis of Andrea Gibson’s poetry and an episode of the HBO TV show *The L Word* (2004–2009), both of which participants discussed. This enables an exploration of how individual texts might function to encourage or enable experiences of connectivity and care through their formal and aesthetic properties such as syntax or imagery. I first discuss participants who found that depictions of self-harm suggested to them a particular method which they went on to use. Their reflections complicate any simplistic idea of a straightforward movement from textual representation to physical harm. Through this, I highlight that care and injury might co-exist rather than being mutually exclusive, and trouble any easy framings that posit care’s primary function as to establish distance from self-harm through silence and avoidance. I then explore experiences of creative transformation and community through engaging with poetry and zine making, and consider the aesthetic properties of poems which might invite such responses. Finally, I discuss ways that repetitive viewing might function as a form of care through making time to engage with the idea or experience of self-harm. Throughout, I note that the textual relationality participants engaged in, through which creative works were transformed and made use of, offers opportunities for being closer to the topic, concept, and experience of self-harm, and that such closeness functions as a form of care rather than injury or harm. Moreover, I emphasise the ways that specific properties of these creative texts enable transformation and relationality through their repudiation of the shame, silence, and cruelty which often surrounds self-harm.

## Contradictions and complex effects

The concern most frequently articulated with regards to depictions of self-harm is that they might prompt readers or viewers to begin self-harming. This was far from the primary frame through which participants in my research assessed fictional texts, and indeed was not a particularly common theme across the interviews. However, there were two instances where participants specifically described that a fictional text provided an idea for a method of self-harm which they then used. These accounts are notable for their nuance and complexity in contrast to dichotomous frames in which texts are straightforwardly good or bad, with singular and universal material effects.

Lou, a bisexual, white British woman in her 30s, discussed a memoir predominantly focused on anorexia. She said “I remember reading in that book, a method that I hadn't thought of, and that then I went on to use […] So I remember reading that this girl had put a glass in a towel and then smashed the towel against the door to keep the glass self-contained. And I was like, did, you know, that's, that, you know, I, I did that. And that was like a practical tip […] Yeah, that was … such a unhelpful thing for me to know, ultimately.” This was not a question of *beginning* to self-harm, but rather involved a new *way* of self-harming. For Lou it was clear that this knowledge of a method was unhelpful, albeit not in the way that commentators or critics generally imagine a depiction of self-harm to be. Yet even so, there was a complexity in what this meant, in how a text might or should respond to this possibility of harm. Lou reflected on the HBO TV series *Sharp Objects* (2018), that she’d found very triggering, but which another friend, who also had experience of self-harm, had really enjoyed. She commented, “you could censor something and create a story that was safe for me but actually might not be in any way safe for somebody else so who's, who holds that responsibility?” Lou’s comments make clear that the movement from text to practice is not inevitable, or inherent within the text: there is something alive and uncertain in the process, whereby what one person takes from a text, the way it acts in their life, might be radically different from another person. Care and harm emerge as difficult to extricate from one another or to clearly delineate.

Sally, a queer, white British woman in her late teens to early 20s, talked about watching the Channel 4 TV show *My Mad Fat Diary* (2013–2015). She really liked the show because it sensitively and thoughtfully portrayed a fat female protagonist which she hadn’t really seen before. She also liked the show’s approach to mental health, saying that “I felt like it wasn't, um glamorized […] So I was really glad about that.” However, she went on to note that she watched it at a time when she was self-harming, and she remembered that “there’s this one moment where she turns the shower on really high. And I immediately thought, that’s an easy way, d’you know? Like, as in I immediately thought that’s so much easier than what I’ve been doing. And so in that way, it actually did inspire me.” She said she went on to copy that method of self-harm, even though she hadn’t deliberately watched the TV show in order to seek out methods of self-harm.

She had a very clear sense both of the show’s quality, as a thoughtful and realistic portrayal of mental health, and of the show’s positive impact in her own life with regards to body image. She said that the “predominant” feeling upon watching it was that of “relief and a release in itself because you’re like, *Oh, wait, this girl is really funny, really likable*. […] It’s really affirming.” She identified this as a contradiction, saying “how does something that literally did cause me to harm myself but also caused me to feel seen? […] I was like, wow, this really like, it's how it feels like so close to my story. But on paper, it hasn't helped me, d’you know what I mean, so, um, that, that's quite a blurred line.”

This makes clear that care, or an object’s potential to offer or be used as care, does not only exist in the absence of harm. In Sally’s experience, harm and care are not mutually exclusive; they are interwoven, sometimes in ways that are hard to make sense of. Literature on care has, in recent years, been at pains to establish some of care’s more contradictory or thorny aspects. STS scholars Vincent Duclos and Tomás Sánchez Criado have expressed concerns regarding the way care can come to stand in for “shared desire for comfort and protection” and thus be complicit with desires to avoid problematic encounters, functioning as avoidance or conservatism (2020, 153). However, these critiques primarily foreground the potential for discourses of care to be mis-appropriated in the service of cruelty and harm, or for harm to be mis-read as care; they are less interested in the possibility for genuine care and genuine harm to co-exist or to intertwine. Yet this is a possibility that is particularly significant in the context of self-harm, which has been variously understood as attention-seeking, as a form of self-destruction, or as an inappropriate or failed coping mechanism. These are all framings which take for granted the harm of self-harm, that it is a bad or maladjusted practice. However, sociological framings have recently begun to understand self-harm not only as a form of potentially productive bodywork (Chandler, 2016; Gurung, 2018), but even as an act of self-care. Sociologists Zou Simopoulou and Amy Chandler, analysing the accounts of young people, suggest that through the claiming or recognition of the hurt body “as ‘something that is theirs only’ […] self-harming comes to resemble a kind of self-loving” (2020, 114).

Here harm and care come to exist in complex relation; if this is the case for self-harm itself, then perhaps it is unsurprising that fictional depictions of self-harm might, too, engender complex effects whereby harm and care are not easily disentangled. These accounts highlight that simply avoiding or erasing the depiction of self-harm in fiction does not guarantee the presence of care (it is notable that this is not a solution offered by either Lou or Sally), and that the depiction of self-harm, even if it contributes to self-harm being taken up as a material practice beyond the text, is not straightforwardly care-less. Care is uncertain, and contradictory, and its meaning and presence cannot be dictated or determined by any easy assumption.

## Transformation and relation through creativity

Two participants talked about the ways that they engaged with texts, particularly poetry, that felt connected with self-harm, and the ways that those texts enabled processes of creativity and relationality through their own aesthetic properties. Tracey was a heterosexual white British woman in her 30s. Looking back at the time when she was self-harming during secondary school, she talked about the way that creating zines (both of her own writing and collating writing from other authors) came to be a source of solace and connection. She said that “writing poetry or making zines and trying to create a different sort of world … in retrospect was about trying to have something beautiful and nice that didn’t feel at all like I felt”. She raised the topic of zines because one had included a poem by art historian Edward Lucie-Smith titled “Red”. For her, the poem resonated with the “really good experience” of seeing blood as part of her practice of self-harm. She said that “the poem about red expressed something that was quite a beautiful and personal experience to me, and it did so in a way that wasn't very obvious”. Tracey’s experience contains an interesting doubling, in which things that might be difficult or painful are repeatedly reframed or transformed into something that could be understood and experienced as valuable, rather than as shameful, bad, or wrong, as self-harm often is assumed to be (Potter, 2011). First, Tracey frames self-harm itself as an experience that could be understood as ‘beautiful’; although self-harm might be entangled with a difficult part of her life, there was something about the act itself that was pleasing, that allowed for something good, specifically through seeing blood. Lucie-Smith’s poem then took this experience where she found visual beauty and articulated it through an affective, creative, and aesthetically pleasing form through skilfully chosen words and compelling phrases. Finally, a third time, Tracey herself transformed the poem from merely something contained within an art schoolbook to an object compiled amongst other significant texts, through which she both found solace and expressed her own creativity and taste.

Repeatedly, experience, aesthetics, and active engagement intertwine. A material practice of self-harm is creatively significant, and then the aesthetics of the poem are made significant again through their material shift into a zine, a zine in which they were connected to other aesthetic aspects of Tracey’s experiences. The relation between the poems and Tracey, their aesthetic properties and Tracey’s own creative practice of zine-making, affects a transformation of their significance, their meaning, and their function in the world. Tracey didn’t use the word care, but there was something in her positive description of the zines, even in the tone of the specific language she used (such as the repeated us of ‘beautiful’, the description of it as ‘really good’), that invoked a sense that the zine-making and the engagement with poetry seemed to allow a form of self-care. The nature of this care to me seemed to be a mode of closeness, akin to ‘being with’ or even Haraway’s formulation of care as ‘staying with’ and engaging with topics that are complex and thorny (2016). It was a way for Tracey to engage with her own experiences of self-harm, albeit mediated through aesthetics, through language, through the material practices of zine-making.

Cultural sociologists Ash Watson and Andy Bennet suggest zine-making practices often “give comparatively intense attention to capturing the aesthetic experience of an ultimately trivial experience” (84, 2022). There is a stark contrast here to the content of Tracey’s zines, which were far from trivial, but were culturally dismissed or silenced, in the same way that experiences designated as trivial might be. Tracey described the way that others seemed to find it impossible to talk about her self-harm, avoiding the subject or simply failing to “push things” or respond to visible marks on her body. She imagined her niece of the same age and said “I just can’t imagine the adults in her life not taking care of her”; it is clear that she felt the silence and avoidance as, at least in retrospect, a lack of care. In this context, zines emerge as a way of attending closely to experience which is otherwise marginalised, and through that attention creating a practice of care. This care seems to me to be significantly connected to a ‘closeness’ to self-harm, which encompasses emotional comfort with self-harm as a topic and a concept, and a willingness to engage with it in detail and over a sustained period of time without judgement, disgust, shame, or even a sense of physical, emotional, or intellectual recoiling — a stark contrast to the responses Tracey and other participants frequently received.

This wasn’t the only aspect of zine-making that held significance for Tracey; she talked specifically about the fact that “the zines felt quite social. Because you’d get involved with a network of people who write”. She had talked about moving to a private school when she was 11, saying “it was a very different class background to my own” and it took her a while to make friends. After having initially sent away for zines to read, she began to make her own, “And then a friend of mine at school, we started doing it together, so then it was more of a social thing. And there’s just something quite … I don’t know, it’s fun!”. The zines allowed for connectivity and relationality at a time when Tracey felt isolated. Although she did not mention it, I was struck by the way that self-harm itself is also often an isolating or secret experience (Brown and Kimball, 2013). Zine-making opened up a possibility of being close to self-harm in a social setting, even if obliquely. Zines transformed the experience both of self-harm and of reading poetry from one which was conducted by a single person to one which had a sociality or a community, without the initial practices themselves having to take on a public aspect, or even be explicitly described and discussed.

Tracey’s account resonated with the experiences of another participant, Hattie, who was a queer, white, cis woman in her late teens to early 20s, with experience of mental health problems. She talked about the poems of Andrea Gibson, saying that “I don't think they have any like poems that are like specifically like all about self-harm but it sort of like features in quite a lot of their poems. And like, that's like been a massive, like massively important thing to me. Just like been like a really comforting thing to … just yeah. They’re just really beautiful poems.” Once more the word “beautiful” was used; it was the poems’ ability to be aesthetically pleasing, which brought them significance and value. As in Tracey’s account self-harm is connected with (or reframed as) something that can feel good and be socially and personally valued through the aesthetic quality of the poems in contrast to its frequent association with shame, disgust, and failure (Heney 2022). Here, aesthetics do not function to minimise or flatten pain, as in accusations of glamourisation which were prominent in the broader interview data, which I discuss in detail elsewhere (see Heney 2022). The question of how these two effects of texts differ is not easily articulated, and indeed might vary from person to person; nonetheless, it seems important that these poems were not only notable to Tracey and Hattie because of the accuracy of their content, but also because of their aesthetic quality.

Hattie went on to talk about seeing Andrea Gibson perform live with her sister, saying “it was just the nicest thing. […] Everyone like just sat cross legged on the floor. And it was just … like it just had like the nicest atmosphere ever. It was just like a load of very nice, lovely people, and everyone was really friendly and it was just like this atmosphere and everyone, like literally everyone was just crying and … like, I think it's just kind of knowing that someone like shares that experience, that you're not on your own.” Reminiscent of Tracey’s discussion of zine-making, through her material engagement with Gibson’s poems (by physically attending a reading), Hattie is able to find connection and community in the context of an experience that is often isolating. Hattie, like Tracey, didn’t use the word care, but it seems to be present in the description: that through finding meaning in these poems, and through engaging with them and their creator, it was possible to feel cared for, or perhaps simply to feel that care was possible, in a context in which it so often is not. Once more active engagement with poetry effects a transformation of the feelings associated with self-harm through both aesthetic quality and relationality. In both cases, it became possible to engage closely with self-harm in a way that felt positive, and even caring.

Beyond participants’ active material and embodied practices, there may be aesthetic aspects of these poems which invited or were open to such social engagement, and which establish their ability to engage deeply with the topic of self-harm through aesthetic quality rather than through superficial glamourisation. Taking Gibson’s poetry as an example, I will consider how their use of language and imagery might function as an invitation to community and care. Gibson’s poem “I Sing The Body Electric, Especially When My Power’s Out” (2011) might easily resonate with experiences of self-harm; the poem includes a reference to opened veins that infers self-harm or suicide. In the lines that follow Gibson describes a doctor asking if they did it for attention, a dismissive accusation frequently aimed at those who self-harm, to which Gibson responds (2011, 104):

For the record, if you have ever done anything for attention,

This poem is attention

Title it with your name

It will scour the city bridge every night

You stand kicking at your shadow

Staring at the river

The language is lush and evocative, a pleasant aurality to the phrase “scour the city bridge”. The bridge itself is halfway between located particularity and detached iconicity, the kicked shadow and dark river moving between the metaphorical and the literal, as the poem creates intimacy both through its own specificity and its invitation to the reader to personalise it, to call it by their name. Gibson picks up the word “attention”, a word that seems to have been flung at them, scornfully, painfully, and turns it around, creates from it a sense of care and community. Rather than permitting the traditional framing of attention-seeking as weakness or failure, Gibson finds in it a mode of connection. The term animates the poem, transforming it from words on a page almost into an object with agency, something that follows the reader around and even enlivens the reader’s own materiality, their body, which Gibson invokes to claim the poem “does not want to find your body / doing anything but loving what it loves”. Here, within the poem, they seem to be attempting exactly what Tracey and Hattie identified as significant in the aesthetic properties of the poems they valued; that something painful, isolating, solitary, is transformed into a possibility for connection, for beauty, for love.

In the final stanza Gibson talking about singing “what hurts” (2011, 105):

And the echo comes back

Bless your heart

Bless your body

Bless your holy kneecaps

They are so smart

A true benediction, one that allows readers to both seek the poem’s blessing and to receive it, just as the poem itself seems to simultaneously offer out the words and to hear them, an echo within the poem and beyond. The poem draws together distress, beauty, care, intimacy, connection, and materiality; even before it is read, before it offers itself to the reader as something that might be taken up and made use of, that might be made-material in a myriad of ways, that might perform a function of care. The poem uses syntax and imagery to invite the reader into active relationality and engagement, and through such engagement into a mode of care as closeness to difficulty, pain, and even self-harm.

## Return and repetition: Making time for care

Making active use of a creative text to make care possible in relation to self-harm was also present in my discussion with Francesca, a queer, mixed race, disabled woman in her 30s. In talking about Showtime’s *The L Word* (2004–2009), she discussed a specific scene in which the character of Jenny self-harms and then is interrupted and cared for by her friend, Shane.

I used to look that scene up online and like watch it over and over […] I think I would watch it when I was in the same kind of state that I would be in if I was going to actually self-harm. So I think in a way, it was a way of kind of going through that process without having to actually do it, you know? […] I think in some ways, like, it probably was a bit of like a replacement for it, you know?

Many participants in the study talked about how certain texts came to be precious or important to them, and even described returning to texts several times. However, this instance stood out to me in part because what was returned to was not an entire text, or even an episode of a TV show, but a single scene. The scene was drawn out from its context, becoming almost a text in its own right, but also becoming object-like. While still somewhat immaterial, merely a string of code hosted by a video website, in Francesca’s account it seems increasingly thing-like, taking on a solidity in the way it can be accessed at will, made use of. There is an echo here of the zine-making practices Tracey described, of the way a creative text can be extracted and transformed or re-purposed.

Francesca’s interpretation of and emotional connection (one might say closeness) to this scene and to the character of Jenny was certainly not universal, nor even shared by all viewers with experience of self-harm, as Francesca herself acknowledged. Even within this small study, there was another participant, Riley, who had watched *The L Word* and talked about finding Jenny’s character very frustrating or annoying. The scene depicting Jenny’s self-harm is precious to Francesca not solely because of its significance within the TV show; rather it is significant in its relation to her own life and in relation to Jenny’s wider cultural positioning:

“I think if it was just a scene where someone self-harms, and that was the end of it, then I don't think I would watch it again and again. I think it’s because someone self-harms and then someone comes in and cares for them. […] Because obviously, I have had moments like that with people who care for me, but I've also had, like the whole spectrum of reactions that were nowhere near as, you know, helpful. […] And I think like on a bigger scale, because I identified so much with Jenny. And because there was such hatred towards her character in the sort of wider fan community. And because Shane was the most favourite character […] it was like, she was saying, *No, look, she’s okay. We can care for them. We can love her.*

These are contexts wherein certain subjects, perhaps specifically those who self-harm, seem beyond care; they are hated, they receive a poor response to their pain and their distress. Shane’s actions are significant not only because they are caring, but specifically because they are caring in the context of care’s absence; care’s absence in the popular reception of Jenny, care’s absence in the broader social understanding of self-harm, but also perhaps care’s absence in Francesca’s own life. This echoes strongly the experiences of Tracey and Hattie; repeatedly, the transformative potential of active engagement with creative texts takes on significance because of the specific context of self-harm. If self-harm is a practice which does not easily inspire care from others, then textual objects, and their uncanny ability to contain and transmit human relationality or sociality, even when consumed alone, come to take on new significance. The scene itself is an act of transformation, a reframing of how Jenny might be responded to both within and beyond the text and a shifting of the emotions associated with both her and with self-harm. In returning to the text, in reaching out to it over and over again, Francesca multiplies this transformation, finds ways to literalise it beyond the realm of the fictional. The scene becomes an object which can be used to enact care when care is otherwise absent; it becomes precious and personal, but is so because of how Francesca connects it to the social and the interpersonal, because of what forms of inter-relational care it suggests might be possible even they are not yet present.

Yet this is not to say that the text’s ability to function as an object of care lies entirely in the process of interpretation. There are aspects of the scene, and its broader context within the TV show, which perhaps make it particularly open to this form both of emotional investment and of re-purposing. Interestingly, I would argue that it is the show’s weaknesses as well as its strengths which invite these modes of viewing, in contrast to the significance of poetry’s aesthetic quality in Hattie and Tracey’s accounts. Participants in the study often critiqued a representation’s failure to locate self-harm within the context of a person’s life, or within the wider narrative; they disliked when self-harm was used as shock value or simply to further the plot. In many ways, *The L Word* entirely falls victim to this trend. While Jenny’s distress and emotional instability are well-established, her self-harm is essentially unmentioned outside of this singular episode, the finale to the show’s second season. It is unsurprising that Francesca identified the scene as containing within it an entire emotional arc; this singular scene is in many ways the entirety of the show’s narrative about self-harm, and is therefore particularly open to being consumed as a self-contained text. This strange detachment perhaps invites the sort of active meaning-making response which Francesca engaged in, as the sequence invites viewers to contextualise it themselves —– whether within their understanding of Jenny’s life, or indeed within their own lives.

The show makes no use of a soundtrack to heighten the tension of the scene; the sounds Jenny makes as she takes out the razor blade and the dialogue exchanged with Shane both seem more stark against the silent background. This is echoed in the lighting of the scene, which is neither softened nor aestheticized; the light is bright and the walls of the bathroom are painted white, there is nowhere to hide. The dialogue, too, is minimalist; the scene is emotionally intense but not melodramatic. When Shane sees Jenny, she doesn’t remonstrate or ask for explanations; instead she reassures, she says “it’s alright”, and “we’re going to get you help”, to which Jenny acquiesces “I need help”. The scene doesn’t manufacture an emotional release; indeed, it seems almost understated. The dialogue is abstracted from the specifics of the characters and the scene, perhaps also encouraging audience members to connect the scene to their own experiences, to find intimate or personal relation to the unfolding interaction. Moreover, in Shane’s tenderness, her calmness, it is easy to see how Francesca might find comfort or even care in the scene’s narrative arc. Yet this calmness itself is notable in part because of what proceeds or surrounds it; the sound of Jenny’s wrenching cries as the camera focuses on Shane in the hallway, the explicit sight of the cuts Jenny’s made on her thighs, her very evident distress. In the quote, Francesca emphasised the importance of the care Shane is able to show, noting that simply a scene of self-harm wouldn’t provide comfort in and of itself. Yet the comfort wasn’t separate from the pain; this wasn’t a depiction only of care, it was a depiction of intense distress which was met with care. In part, this reflects general aesthetic theories of catharsis, in which emotional tension and resolution invoke emotions that are then released, in a process widely acknowledged as at least meaningful, and even potentially therapeutic (Scheff, 1979). However, I would suggest that there is also a specificity with regards to the subject matter that encourages emotional engagement and intensity. There is an echo here of the contradictions of Sally and Lou’s accounts, in which care and harm are not separate; the care is meaningful because it follows the particular pain of self-harm, because it allows that pain to come into contact with care in a way in which it might not usually.

Francesca said, explicitly, that “I think I would watch it when I was in the same kind of state that I would be in if I was going to actually self-harm. So I think in a way, it was a way of kind of going through that process without having to actually do it, you know?” Although she framed the experience in terms of affectivity and emotional state, her comment also, to me, invoked a question of temporality. She watched the scene at the *time* when she might have self-harmed, that the video was a way of spending that time wherein the concept or experience of self-harm was still invoked, or brought close, but through the scene was simultaneously aligned with an experience of care. There is a complex relation here between substitution and avoidance; in part, the text stands in for self-harm, it allows a feeling of pain followed by catharsis that might otherwise be found in self-harm. But it does so not by simply avoiding or ‘resisting’ self-harm, denigrating it as a flawed or foolish coping mechanism. Rather, Francesca’s practice of repeated viewing recognises what is meaningful about self-harm, and engages deeply with it over time.

The question of time prompts a consideration of de la Bellacasa’s insight that care requires ‘making time’ of a ‘particular kind’ (2017, 206). Logics of absence and silence suggest that self-harm is best cared for by spending as little time as possible engaging with its representation. However, in the experiences of Hattie, Tracey, and Francesca, it is precisely time spent engaging closely with the concept and experience of self-harm that feels like care. The text itself creates time, the time required to read and experience it, but in returning to it, in attending a poetry reading, or making a zine, or finding a YouTube video, the text is made meaningful through time, and time is extended through a process of active engagement with the text. Within and through this time, the aesthetic qualities of texts invite and enable new relationality and affectivity around self-harm, encouraging or making possible active engagement that transforms that which is solitary and shameful into something associated with beauty and creativity. Self-harm is turned from something that must be avoided through silence, into something it is possible to be close to, to spend time with – beyond (and indeed separate from) actually self-harming. It is in this relation, between time, textuality, engagement, and self-harm, that care is made possible, perhaps against all odds.

## Conclusion

This paper offers a reading of several small fragments of data, and of two accompanying texts, in order to articulate a possible relation between creative and fictional texts, care, and self-harm. I do not, by any means, suggest that they are able to entirely illuminate the way that creative texts function as objects-of-care in the context of self-harm; rather I hope that I have been able to explore facets of the dynamic relation between text and self through which aesthetic properties come to form or contribute to modes and practices of care. By moving between analysis of both interview data and creative texts themselves, I hope I have been able to attend to the uncertain connection between text and individual, to the active processes of meaning making which occur in that space, and to the care which can emerge there. I have outlined the way in which this care is not predetermined, singular, and universal, that a relation of care can be invited by aesthetic qualities such as syntax, imagery, and abstraction, the way that creative engagement around texts can create social relations of care, and the way that texts can invite investments of time and attention that can itself be a form of care. Throughout, I have noticed the way that participants understood care as emerging from an emotional and conceptual closeness to the topic or experience of self-harm that texts enabled or encouraged. Thus, I offer an understanding of care (echoing existing theorisations by Haraway and Stevenson) as relational, and as intertwined with difficulty, pain, and harm. However, there remain significant questions about how to determine or delimit what care is, specifically in the context of self-harm. Similarly, I have offered some initial suggestions regarding the ways that creative texts might open themselves to transformational use, through features such as syntax, imagery, and abstraction, but this too remains an area that would merit further study.

While this paper has emphasised the function of active, material, and temporal engagement with texts, the care participants described has often been primarily affective. This is not to suggest that material care is not required or desired; rather, for me, this raises questions about the limits and uncertainties of the care provided by and through creative texts. It is overly optimistic or idealistic to expect fiction to do the material work of care; the work of tending to wounds, of holding hands, of keeping an individual safe in a crisis, of providing psychological support and treatment. Care cannot exist solely in the creative realm; we all need the under-valued forms of tangible care, which people from marginalised groups are so often burdened with providing (for unjust wages and within exploitative working conditions), and which they themselves are often in turn denied (Hankivsky, 2014). Fiction and literature cannot carry the burdens of our failings in care; failings which in the context of self-harm include cruel and punitive treatment in healthcare settings (Faulkner and Rowan Olive, 2022), the threat of criminalisation (Pring, 2021), and a desperate lack of resourcing leading to lengthy waiting lists and inadequate service provision (Make Space, 2023). Fiction cannot be substituted for or resolve these needs or these cruelties; but it can bring us close to self-harm, it can bring connection, and it can help us to make time for care, in the complex and contradictory forms it often comes in.

## Data Availability

Data is available from the author upon request.
