# Supplementary material for: Creating care for people who self-harm through transformation of aesthetic objects
Source: J Med Humanit. Author manuscript; Available in PMC 2025 May 6. (PMC7617644; doi:10.1007/s10912-025-09941-w)
Supplement: Supplementary file 1 [file EMS204989-supplement-Title_Page.pdf]

Title Page

Title: Creating care for people who self-harm through transformation of aesthetic objects

Author Information:

Veronica Heney

Institute for Medical Humanities, Durham University, UK.

Veronica.heney@durham.ac.uk

Orcid ID: 0000-0002-5497-7656

Abstract

The role of fiction in enabling care for people who self-harm is primarily framed as a relation of protection through absence or avoidance. It is frequently suggested that fiction should avoid depicting self-harm, lest it encourage readers to begin self-harming, framing those who self-harm as passive and in need of protection. This paper will demonstrate that when the perspectives of people who self-harm are centred in the analysis of texts and their effects, the practice of reading and viewing fiction emerges as a more active, creative, and relational experience, which brings self-harm close rather than holding it at a distance. Indeed, such active engagement through material practices like zine-making, event attendance, and repeated viewing of a singular scene is understood as that which makes care possible.

Through a novel interdisciplinary approach this brings together sociological and literary methods to explore the dynamic relation between a text and its effects. Drawing on both qualitative interviews with people with experience of self-harm and close readings of creative texts including the Showtime TV series *The L Word* (2004-2009) and Andrea Gibson's poem 'I Sing The Body Electric, Especially When My Power's Out' (2011), the paper traces trace the complex relation between texts and the care they make possible. Thus, I extend existing theorisations of care as intimate and relational to the context of self-harm. Specifically, I outline the way in which care is not predetermined, singular, and universal, and explore the ways that a relation of care can be invited by aesthetic qualities.

Keywords:

Care, Narratives, Self-harm, Interpretation, Reading Practices

Statements and Declarations

The author has no competing interests to declare that are relevant to the content of this article. This work was funded by the Wellcome Trust (Wellcome Grant Number 203109/Z/16/Z, Wellcome Grant Number 221747/Z/20/Z, and Wellcome Grant 226798/Z/22/Z).

For the purpose of open access, the author has applied a CC BY public copyright licence to any Author Accepted Manuscript version arising from this submission. I would like to thank the editors and anonymous reviewers for their careful guidance and constructive suggestions.

This project was reviewed and received ethical approval by the Ethics Committee of the Department of Psychology, College of Life and Environmental Sciences at the University of Exeter. The study was performed in accordance with the ethical standards as laid down in the 1964 Declaration of Helsinki and its later amendments or comparable ethical standards.

Data is available from the author upon request.
